# Performance of the microINR Point-of-Care System Used by Self-Testing Patients: A Multicenter Clinical Trial

**DOI:** 10.1055/s-0041-1740962

**Published:** 2021-12-30

**Authors:** Majed A. Refaai, Alan K. Jacobson, Jack C. Rosenfeld, Robert R. Orr

**Affiliations:** 1 Department of Pathology and Laboratory Medicine, University of Rochester Medical Center, Rochester, New York, United States; 2 Department of Internal Medicine, VA Loma Linda Healthcare System, Loma Linda, California, United States; 3 Green and Seidner Family Practice Associates, Lansdale, Pennsylvania, United States; 4 Phoenix Medical Research, Peoria, Arizona, United States

**Keywords:** international normalized ratio, warfarin, point-of-care systems, self-testing, anticoagulation monitoring

## Abstract

**Introduction** Anticoagulation monitoring is a major practical and clinical challenge. We assessed the performance of the microINR system in patient self-testing (PST).

**Methods** This study was performed at four US medical centers. After the training visit of warfarin anticoagulated patients ( *n* = 117) on microINR system, PST was performed at home and in two visits to the medical centers. At the medical centers, both PST and healthcare professionals (HCPs) performed duplicate tests with the microINR System. A venous blood sample for the laboratory testing was also extracted. Accuracy and precision were assessed.

**Results** The comparison between microINR PST results and microINR HCP results revealed an equivalence with a slope of 1.00 (95% confidence interval [CI]: 1.00–1.00), and an intercept of 0.00 (95% CI: 0.00–0.00). When compared with the laboratory analyzer, microINR PST results also showed good correlation with a slope of 0.94 (95% CI: 0.86–1.04) and an intercept of 0.14 (95% CI: -0.09–0.34). Predicted bias values at international normalized ratio (INR) 2.0, 3.5, and 4.5 were 0% against HCP and ≤2.5% against the laboratory. Analytical agreement with both HCP and laboratory was 100% according to ISO17593 and 99.1 and 100% according to CLSI POCT14 with HCP and laboratory, respectively. Clinical agreement with HCP regarding 2.0–4.0 as INR therapeutic range was 98% (within range). The precision (coefficient of variation) of microINR system used by PST was comparable to HCP.

**Conclusion** The microINR results when used by self-testing patients show satisfactory concordance to both HCP results and laboratory analyzer. The microINR system is adequate for self-testing use.

## Introduction

Vitamin K antagonists (VKA), such as warfarin, are commonly used oral anticoagulants for thrombotic disorders. The anticoagulation effect of VKA therapy is monitored by the international normalized ratio (INR) method of reporting the prothrombin time. The traditional method of INR monitoring consists of a venous blood sample obtained at an anticoagulation clinic or other healthcare facility for the measurement by a laboratory system. Dosage adjustment, if needed, will then be arranged by the healthcare providers (HCPs). INR point-of-care (POC) instruments evolvement reduced clinic visits, provided more testing options and locations, and allowed immediate clinical evaluation and dosage adjustment. Furthermore, the patient self-testing (PST) model, where patients perform INR testing by themselves and report results to a HCP, provided significant advantages to warfarin anticoagulation monitoring. Additionally, the patient self-management (PSM) model upon which patients perform by themselves both INR testing and the dose adjustment augmented INR POC benefits. In comparison to the other regular monitoring models, PST and PSM models showed significant decrease in thromboembolic and bleeding events and provided better clinical outcomes. 1 2 3 4 5 This is mainly due to the improvement in the time in therapeutic range (TTR).

These models are convenient for challenging patients such as those with mechanical heart valves, and particularly in the elderly, as well as individuals who travel frequently. For the elderly, it can be difficult to visit a healthcare facility for routine blood draws at the frequency needed for effective management of VKA therapy, and for travelers, access to venipuncture is problematic. It has been demonstrated that PST and PSM could be an effective and safe alternative to direct oral anticoagulant (DOAC) therapy. 6 They can also be more cost-effective than conventional methods including DOAC therapy due to the reduction of adverse events, *inter alia* . 5 7 8 9 Both models enable the management of anticoagulation therapy to join ongoing trends in telehealth. 10 Literature clearly supports the use of self-testing methods for INR monitoring. However, high-quality studies assessing the diagnostic value of point-of-care testing (POCT) coagulometers in terms of accuracy and precision when self-testing are scarce. 11 12 The microINR system (iLine Microsystems, Donostia - San Sebastián, Spain) is a POC system for INR measurement in patients undergoing VKA therapy that can be used for the self-testing purpose. The specific characteristics in the design and in the use of the microINR system, such as the automatic strip lot identification and calibration, the low sample volume and the minimum testing steps, provide advantages over other POC systems for lay-users. The microINR system was granted an U.S. Food and Drug Administration (FDA) clearance for professional use (K180780) in January 2019, and recently, for self-testing use and for professional use in The Clinical Laboratory Improvement Amendments (CLIA) waived settings (K201185) in December 2020. In a previous study, we demonstrated adequate precision and accuracy to a laboratory system and to another portable INR device, and thus reliability for the management of warfarin therapy by professional users. 13 Here, we present the outcome of a multicenter study that was conducted to assess the performance of the microINR system by self-testing patients under real-life conditions.

## Materials and Methods

This study was performed in different US medical centers between September 2019 and February 2020. VKA-treated patients who were followed up for anticoagulation therapy at anticoagulation clinics, medical practices, and/or outpatient settings were recruited. The aim of this clinical trial was to assess the accuracy of the microINR system in PST versus the HCPs and a reference laboratory method.

### The microINR System

A total of 149 microINR analyzers tagged as “For investigational use only” and also microINR chips (test strips) were provided by iLine Microsystems throughout the clinical trial. Each lot of microINR chips is calibrated to a reference lot of human recombinant thromboplastin traceable to the World Health Organization (WHO) International Reference Preparation.

### Study Design

The study was performed at four US clinical sites; site 1, VA Loma Linda Healthcare System (Loma Linda, California; VA, Veterans Affairs); site 2, University of Rochester Medical Center (Rochester, New York); site 3, Green & Seidner Family Practice Associates (Lansdale, Pennsylvania); and site 4, Phoenix Medical Research (Peoria, Arizona). The study protocol complied with the Helsinki II declaration and was approved by the corresponding ethics committees. All patients enrolled in this study were above 18 years of age and were anticoagulated with warfarin for at least 6 weeks. None of the enrolled patients had prior experience with the microINR analyzer. Patients transitioning from or to non-VKA anticoagulants, known to have antiphospholipid syndrome, or those who had participated in an interventional clinical trial within 1 month of the enrollment were excluded.

### Study Visits

The study consisted of a training visit and two clinic visits ( Table 1 ). At the training visit, the study procedures were carefully presented to all participants along with the consenting process. During this visit, patients were also trained on the use of the microINR system using the instruction manuals and package inserts provided with the analyzers and chips. Once the patient felt confident with the usage of microINR system, an initial assessment questionnaire was then required to be completed. A score of 70% or above demonstrated patient's ability to use the system. Patients included in the study were then asked to test themselves at home for 2 weeks (two self-tests per week). After each week, a visit to the clinical site was scheduled. During these visits, patients performed two self-tests in front of the HCPs using single-use 23 G lancets. In addition, the trained HCPs performed two tests in the same microINR system also using single-use 23 G lancets for the fingersticks. Of note, different fingers were used for each test performed. During the second visit (i.e., last visit), the self-testing patients filled out a questionnaire with 20 statements about the ease of use, handling and functionality of the microINR, rating each statement from 1 (strongly disagree) to 5 (strongly agree).

**Table 1:** Study visits

Visit	Procedures
Training visit	Consenting, training, initial questionnaire, answering any concerns, handling of device and few chips for home self-training, and scheduling of the next 2 clinic visits
First clinic visit	Within 7 days; performing of 2 INR testings by the patient (PST) in front of HCP and 2 INR testings by the HCP. Handling of few more chips as well for the upcoming week
Second clinic visit	Within 7 days; performing of 2 INR testings by the patient (PST) in front of HCP and 2 INR testings by the HCP, and collecting of venous blood sample, final questionnaire, and returning device

Abbreviations: HCP, healthcare professional; INR, international normalized ratio; PST, patient self-testing.

### Blood Samples and Laboratory Tests

During the second visit, a venous blood sample was collected and processed in accordance with the CLSI guideline H21-A5. 14 Two venous blood samples were obtained via venipunctures using a 21 or 23 G gauge needle into an ethylenediamine tetraacetic acid (EDTA) tube (Becton-Dickinson, Oxford, UK, and Greiner Bio-One, Kremsmünster, Austria) and 3.2% sodium citrate tube (Becton-Dickinson, Oxford, UK). The EDTA tube was used for hematocrit testing at each site (site 1, Sysmex XN-9000; site 2, Sysmex XN-9100; site 3, Sysmex XNL; and site 4, Sysmex XN-1000). The citrate tube was processed to plasma, aliquoted in cryovials, and frozen at -80°C within 4 hours of collection. Frozen plasma collected at each site was shipped for reference testing at the centralized laboratory analyzer location (University of Rochester, Rochester, New York, United States). At the time of testing, samples were thawed at 37°C water bath over 5 minutes and tested for INR on the ACL TOP 500 with HemosIL RecombiPlasTin 2G reagent based on recombinant human tissue factor (Instrumentation Laboratory, Bedford, Massachusetts, United States). The calibration of the laboratory system/reagent combination was verified with the HemosIL INR Validate (Instrumentation Laboratory, Bedford, Massachusetts, United States), a tri-level quality control.

### Statistical Analysis

The accuracy of the microINR system was evaluated using the first INR result obtained by fingerstick based on CLSI EP09-A3. 15 A Passing Bablok regression analysis of microINR results by self-testing patients was performed against results by HCPs and the reference laboratory. Predicted bias (%) values at medical decision points of INR = 2, INR = 3.5 and INR = 4.5 were calculated from the regression lines obtained. In the analytical agreement analysis, the percentage of differences within the limits described in Table 2 were calculated for each INR range and assessed. 16 17 18 The clinical agreement was also assessed considering agreement when both the self-testing patients' and HCPs' results were below, within or above the 2.0 to 4.0 INR range of therapeutic levels (the combination of both low intensity group with therapeutic range 2.0–3.5 and the high intensity group with therapeutic range 2.5–4.0). The imprecision of the INR with the microINR system was calculated by determining the coefficient of variation (CV) from the duplicate measurements in accordance with the ISO17593:2007. 16 Outlier detection was performed according to CLSI EP09-A3 and ISO17593:2007 for accuracy and precision analysis, respectively. Analyses were performed with R statistical software (version 3.6.1).

**Table 2:** Acceptance criteria for analytical agreement analysis

Guidance	Overall agreement	Allowable difference	INR range
ISO17593:2007 16	≥ 90%	± 0.5	< 2.0
		± 30%	≥ 2.0–4.5
FDA Workshop 2016 17	≥ 95%	± 0.4	< 2
		± 20%	≥ 2–3.5
			> 3.5–4.5
		± 25%	> 4.5
CLSI POCT14-Ed2 18	≥ 95%	± 0.4	< 2
		± 20%	≥ 2–3.5
			> 3.5–4.5
		± 25%	> 4.5–6.0
		± 30%	> 6.0

Abbreviations: FDA, U.S. Food and Drug Administration; INR, international normalized ratio.

To assess the ease to use of the system in hands of the patients, the total average score as well as the distribution of each of the scores was calculated from the ratings in the questionnaire filled in the second visit to the site.

## Results

### Participant Characteristics

One-hundred twenty-one patients met the study inclusion and none of the exclusion criteria and were enrolled in the study following inform consenting. All participants demonstrated good understanding and the ability to use the microINR system. During the study, four patients (3%) had to be withdrawn from the study; three due to warfarin therapy interruption and one more due to personal reasons. The hematocrit values of all patients were within the acceptable range of hematocrit (25–55%), according to the manufacturer package insert. Therefore, the performance of the microINR system was analyzed by a total of 117 VKA anticoagulated patients ( Table 3 ). The mean age of the study population was 70 years (range: 38–89). The most common indications for VKA anticoagulation were atrial fibrillation (47%), deep venous thrombosis or venous thromboembolism (15%), and valvular heart disease (14%).

**Table 3:** Patient demographics

Age	Mean (range)	70 (38–89) years
Indication for anticoagulation	Atrial fibrillation and flutter	47.0%
	Venous thromboembolism	15.4%
	Heart valves and devices	14.5%
	Myocardial infarction and myocardiopathy	2.6%
	Other	20.5%
Educational level	High school	35.9%
	University and college degree	33.3%
	Professional degree, Masters and PhD	17.9%
	Vocational, technical, diploma and certificate	6.8%
	Middle school	0.9%
	Other	5.1%
Trained on another INR POC	No	72.6%
	Yes	27.4%

Abbreviations: INR, international normalized ratio; POC, point of care.

### Agreement between Self-Testing, Healthcare Professionals, and Laboratory System INR Values

**Fig. 1:**
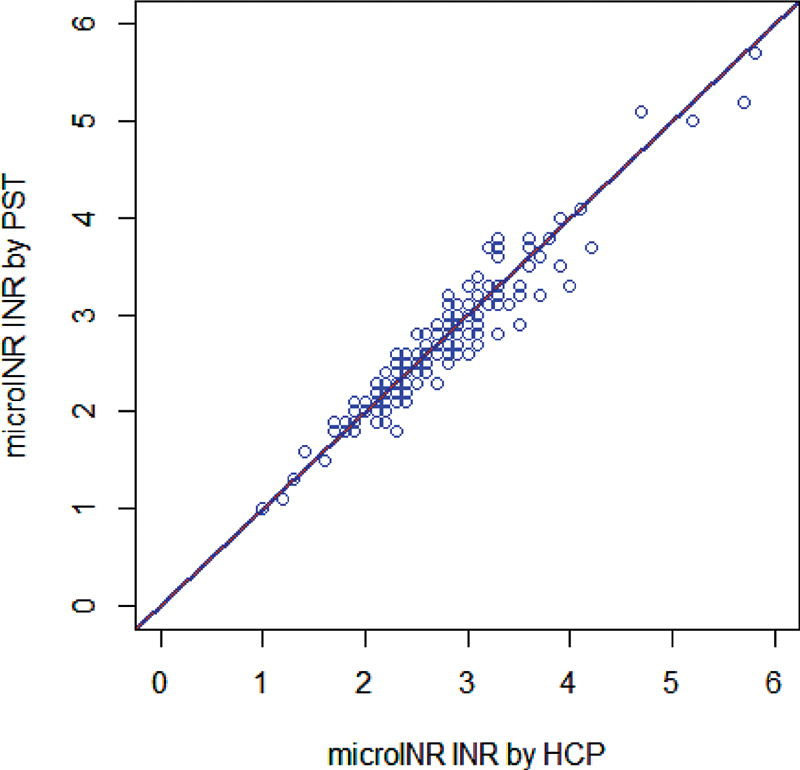
Correlation between self-testing patients' and healthcare professionals' microINR first results of the two visits. Number of samples is 228; Correlation line (continuous): correlation coefficient ( *r* ) 0.955, slope 1.00 (95% CI: 1.00–1.00), Intercept 0.00 (95% CI: -0.05–0.00). Identity line (dotted): y = x. CI, confidence interval; HCP, Healthcare professional; INR, international normalized ratio; PST, patient self-testing.

First INR values that were obtained from both self-testing patients and HCPs with the microINR system and from the laboratory method were analyzed. The regression analysis of the self-testing patients against the HCPs during the two visits to the sites, a comparison of 229 tests, revealed a total equivalence with a slope coefficient of 1.00 (95% confidence interval [CI]: 1.00–1.00) and an intercept of 0.00 (95% CI: 0.00–0.00), and a correlation coefficient ( *r* ) of 0.952. The outlier detection analysis, by performing the method described in CLSI EP09-A3, identified only one INR value as an outlier between self-testing patients' results and HCPs' results (2.6 vs. 3.3 INR, respectively). After the removal of the outlier, the *r* increased from 0.952 to 0.955, with no change on the slope and the intercept values ( Fig. 1 ).

**Fig. 2:**
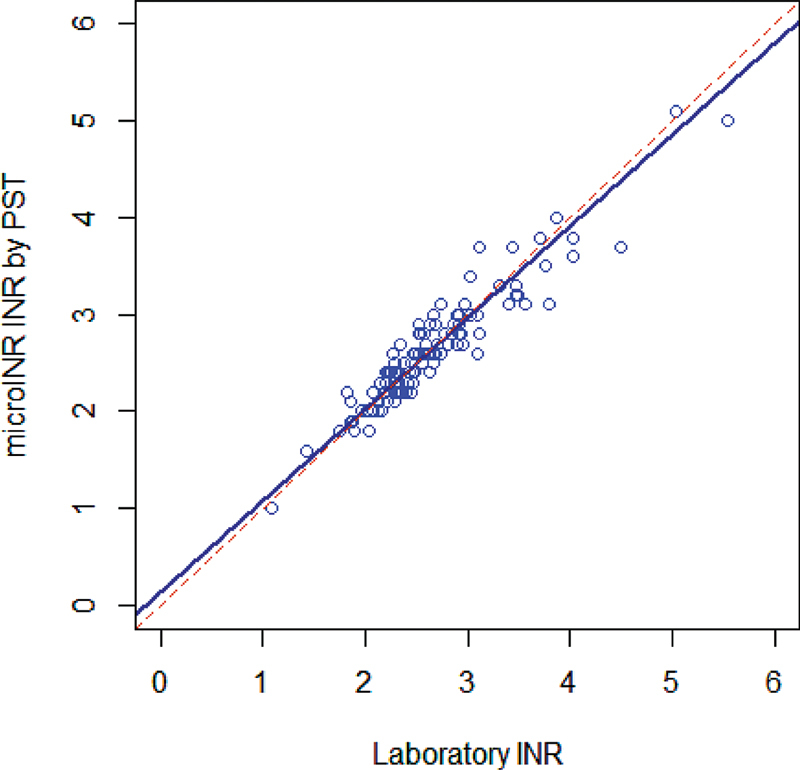
Correlation between self-testing patients' microINR first results and ACL TOP 500 of the Visit 2. Number of samples is 114; correlation line (continuous): correlation coefficient ( *r* ) of 0.943, a slope coefficient of 0.94 (95% CI: 0.86–1.04) and an intercept of 0.14 (95% CI: -0.09–0.34). Identity line (dotted): y = x. INR, international normalized ratio; PST, patient self-testing.

The regression analysis of the self-testing patients' results with the microINR system against the laboratory system also showed remarkable agreement with a slope coefficient of 0.94 (95% CI: 0.86–1.04) and an intercept of 0.14 (95% CI: -0.09–0.34), and a correlation coefficient ( *r* ) of 0.943 ( Fig. 2 ). No outliers were detected.

Table 4 shows the predicted bias at medical decision points of the self-testing patients' results against HCPs' results and laboratory system results. Predicted bias values at INR 2.0, 3.5, and 4.5 were 0% against HCPs and ≤2.5% against the laboratory.

**Table 4:** Predicted relative bias results of microINR system used by self-testing patients against microINR system used by healthcare professionals and ACL TOP 500 system

		INR = 2.0	INR = 3.5	INR = 4.5
vs. healthcare professionals	Predicted bias %	0.0	0.0	0.0
	95% CI:	(0.0, 0.0)	(0.0, 0.0)	(0.0, 0.0)
vs. ACL TOP 500 system	Predicted bias %	1.4	−1.6	−2.5
	95% CI:	(−1.2, 4.5)	(−4.7, 1.3)	(−6.7, 1.7)

Abbreviations: CI, confidence interval; INR, international normalized ratio.

The overall analytical agreement between self-testing patients' and either HCP's results ( *n* = 229) or laboratory results ( *n* = 114) reached 100% according to the acceptance criteria of the ISO17593:2007. If analyzed according to the criteria proposed by FDA in the 2016 workshop or the recently released CLSI POCT14-Ed2, an overall agreement of 99.1% was met when self-testing patients' results were compared with HCP's results and 100% when compared with laboratory results.

### INR Therapeutic Levels Agreement between Self-Testing and Healthcare Professionals Values

In the INR range of therapeutic levels (INR 2.0–4.0), clinical agreement between self-testing patients' and HCPs' results was in 98% of the results ( *n* = 202). At subtherapeutic INR levels (INR <2.0) and at supratherapeutic levels (INR >4.0), the number of patients was lower and clinical agreement resulted in 82% ( *n* = 22) and 100% ( *n* = 5), respectively. The overall clinical agreement was in 97% of the results.

### Precision of the microINR System

The microINR system's imprecision (repeatability) by self-testing patients and HCPs was determined by quantifying the random error/variation within duplicates in terms of CV. The overall CV of the microINR by self-testing patients in the two visits to the sites was 5.4%, and noticeably, it decreased from first visit to second visit, from 5.9% to 4.9%, approaching the result obtained by professionals ( Table 5 ). The overall CV of the microINR by HCPs was 4.6%. If statistical outliers were excluded from the data analysis, the CV for self-testing patients in visit 1 was 5.1%, and the overall CV were 5.0 and 4.2% for self-testing patients and HCPs, respectively.

**Table 5:** Imprecision of the microINR system

	Self-testing patients			Healthcare professionals		
	*n* test pairs	SD	CV%	n test pairs	SD	CV%
Overall	214	0.14	5.4	221	0.12	4.6
Visit 1	103	0.15	5.9	110	0.11	4.3
Visit 2	111	0.13	4.9	111	0.12	4.5

Abbreviations: CV, coefficient of variation; SD, standard deviation.

Four statistical outliers in the self-testing patients' and one in the healthcare professionals' results were detected. If excluded, the CV for self-testing patients in visit 1 is 5.1%, and the overall CV results are 5.0 and 4.2% for self-testing patients and healthcare professionals, respectively.

### Participant's Assessment of the microINR System

With regard to the ease of use of the system, during the second visit to the clinical sites, 96.1% of the statements in the questionnaire were rated with 3, 4, or 5 by self-testing patients resulting in an overall average score of 4.7 out of 5. The highest satisfaction averages were reported for “Easy to read results” (4.92) and “Turning on the Meter is easy” (4.87). The administered questionnaire and patients' average scores per statement are given in Table 6 .

**Table 6:** Questionnaire administered to the patients for self-testing assessment at home during the second clinic visit

	Statement (S)	Mean score
S1.	The symbols and numbers that appear on the Meter screen are easy to read and understand	4.74
S2.	I liked the size of the Meter and the Meter button	4.71
S3.	It is easy to understand when to apply the drop of blood	4.70
S4.	The Chip is easy to manipulate	4.41
S5.	The amount of blood sample needed is easy to obtain from a fingerstick	4.62
S6.	I understand how I should store the Chips	4.81
S7.	The result is easy to read	4.92
S8.	The meaning of the result is easy to interpret	4.80
S9.	The time it takes for the Meter to give a result is not too long	4.38
S10.	The procedure to perform the test is easy to learn	4.72
S11.	Using the Meter is easy to learn	4.74
S12.	Turning on the Meter is easy	4.87
S13.	Opening the Chip package is easy	4.19
S14.	Inserting the Chip into the Meter is easy	4.50
S15.	I was able to apply blood to the test strip within 80 seconds of lancing the finger	4.79
S16.	It is easy to remove the used Chip	4.80
S17.	It is easy to see the results in the memory	4.85
S18.	It is easy to identify the meaning of the error messages in the instructions for use	4.68
S19.	The “EasyGuide” is clear and useful for understanding the operation of the microINR system	4.73
S20.	The instructions for use are clear and useful for understanding the operation of the microINR system	4.77

## Discussion

The performance of the microINR system was recently evaluated in a multicenter study in a professional setting. 13 The positive results obtained from the initial study prompted us to further test and evaluate this POC system in a self-testing setting by lay users. All the results of accuracy and precision obtained in this study by HCPs and by self-testing patients with the microINR system are consistent with the results obtained by professionals in the previously reported study. 13

In comparison to the HCPs, INR results of the microINR system obtained by self-testing patients showed equivalence, in terms of slope of “1” and intercept of “0,” and no differences at clinical decision points. An overall analytical agreement of 100% was achieved either against the HCP results or the laboratory results based on ISO 17593 acceptance criteria. 16 Additionally, 100% agreement was met between self-testing patient results and laboratory results in accordance with the more restrictive criteria mentioned by FDA at the workshop in 2016 and the recently published CLSI POCT14 guideline. 17 18 Significant agreement (99.1%) is achieved also against HCP results according to both mentioned criteria. Furthermore, the good performance of the microINR system that is perceived with the few INR values over 4.5 INR should be also highlighted. From the clinical perspective, in the present study an overall agreement of 97% (therapeutic INR range set at 2.0–4.0) was obtained between self-testing patients and HCPs showing that same clinical decisions were to be taken from microINR results of either self-testing patients or professional users. 11 This reveals that lay users can obtain similar results as the HCPs, and consequently, significant correlation to a reference laboratory system (ACL TOP 500 analyzer, RecombiPlasTin 2G thromboplastin reagent) as well as good analytical and clinical agreements.

The microINR system when used by self-testing patients showed good overall precision of 5.4%, performed with duplicate capillary blood samples and including the sub- and supratherapeutic INR values. Note should be taken about the 1% decrease in CV between the first and the second clinic visits, from 5.9 to 4.9%. Only some limited practice at home, that is, only two self-tests were performed at home between visits, considerably improved the CV result obtaining a value comparable to the 4.6% CV obtained by healthcare professionals. In general, it is common to perceive a reduction in the CV as the users become familiar with the system. For the CoaguChek XS system, as reported by Braun et al in 2007, the CV was 5.92% at the beginning of the study and 5.16% at the final session after the self-monitoring phase at home. Over the study period, patients gained significant experience in self-monitoring as they had to measure INR eight times in 4 weeks using the CoaguChek S system. 19

In our study, self-testing patients felt comfortable using the microINR system according to the questionnaire they filled out during the second visit. They almost fully agreed on the ease of use (4.7/5) of the system in relation to its simple design, physically and for conducting a test. These results support the idea that the microINR system can be used by patients with different capabilities and age and be properly used for self-testing fulfilling patients' wishes.

Regarding the self-testing patients studied population, all the enrolled patients, with some exceptions, had one or more comorbid conditions (chronic inflammatory conditions, diabetes, high blood pressure, kidney diseases, malignancies, and others) and took concomitant medication(s). Many of them also suffered from different conditions affecting physical, sensory, and cognitive capabilities (e.g., 5.1% suffered from tremors, 16.2% arthritis, 2.6% hearing loss, 8.5% vision impairment). Other factors potentially affecting the comprehension and use of the system, such as educational/cultural level or any previous experience in other self-testing systems (although 27.4% had experience with similar devices, all study patients were naive to the microINR system) were not considered in the inclusion/exclusion criteria so that VKA self-testing population was widely represented in the study. None of these conditions seemed to affect the overall use of the system or biased the results obtained. In addition, the no selection of any kind in the recruitment (100% of the enrolled patients were included in the study after the training visit) and the low practice required by the patient to get a good training level show the robustness of the system for a broad targeted self-tester population. The automatic lot identification and calibration with no need of additional elements, the low volume of blood sample required (at least 3 microliters), the result reported only in INR units, and the simplicity for the testing make the microINR system safe, easy to learn and user-friendly. Nevertheless, this is also reinforced by the acoustic signals and Chip illumination assisting the interaction with the analyzer.

Barcellona et al reported the potential clinical benefits of using the in-home self-testing portable INR POC devices. The foremost advantage was the shifting of INR monitoring from the traditional locations at a thrombosis center to self-testing at home. In addition, the PST and PSM advantages were significant over traditional laboratory methods that include the no need to attend a thrombosis center or anticoagulation clinic in person, avoiding long waits for blood sampling and INR results, and the serenity the self-testing confers. Users were less worried about the possible side effects of VKA therapy because the INR can be measured whenever they feel that some factors may have interfered with VKA treatment or even just for their personal tranquility. 20 In this regard, PST and PSM are very convenient for challenging patients such as those with mechanical heart valves, elderly and traveling patients, and combined with telehealth solutions enable distant care for the outpatient population, so advantageous considered nowadays. 10 21 In addition to these inherent advantages of PST and PSM, the microINR system clearly provides autonomy for the patient and also facilitates its use by caregivers.

In conclusion, the present study shows equivalent performance of the microINR system when used by self-testing patients and by HCPs. The microINR system is a very adequate INR system for self-testing use and can help improve the TTR and clinical outcomes of VKA anticoagulated patients.

## Notes

**What Is Known on This Topic**

- Literature of the performance of portable INR systems by self-testing patients is scarce.

**What Does This Paper Add**

- We performed the first multicenter clinical study of the microINR system used by self-testing patients.
- Study data demonstrate equivalence between self-testing patients' and HCPs' microINR results.
- MicroINR is a reliable warfarin anticoagulation monitoring system for self-testing and professional use at POC settings.
